# Covid-19: a catalyst for cybercrime?

**DOI:** 10.1365/s43439-021-00024-9

**Published:** 2021-04-22

**Authors:** Mohamed Bou Sleiman, Simon Gerdemann

**Affiliations:** 1 Public Prosecutor’s Office Goettingen, Goettingen, Germany; 2 Georg-August-University, Goettingen, Germany

**Keywords:** Corona, SARS-CoV-2, Pandemic, Phishing, Malware, Cyberattacks

## Abstract

While the second wave of the Covid-19 pandemic is keeping the world on tenterhooks, the last few months have also led to a new wave of cybercrime. The following article analyzes the background and manifestations of pandemic-related cybercrimes and shows how our criminal law systems are able to deal with current challenges in the age of the coronavirus.

## Introduction

Various measures taken in countries around the globe to contain the spread of the novel coronavirus SARS-CoV‑2 (Covid-19) have led to public lockdowns and other severe social restrictions. As a consequence, people have relocated many aspects of their private and professional lives out of the physical and into the digital realm. While the restrictions on public life are partly responsible for a decrease in various kinds of “real world” crime, those crimes’ digital cousins have flourished under the new environment. With the spread of the virus and a general sense of uncertainty and insecurity among many parts of the population, Covid-19-related attacks by cybercriminals have become something of a pandemic on their own. International law enforcement agencies such as Europol (e.g. [17]) and Interpol (e.g. [18]) have issued stark warnings about the increased risk of such cyberattacks, which could have issued devastating consequences—especially if they hit critical healthcare infrastructures. Google’s email service alone reported more than 240 million spam messages per day related to COVID-19, as well another 18 million malware and phishing emails being blocked [19]. Likewise, the German Federal Office for Information Security (BSI) has detected a significant increase in cyberattacks related to Covid-19 just shortly after the virus was recognized as a source of an upcoming pandemic [20]. In this regard, not much has changed during the long course of the pandemic, especially after the “second wave” of the pandemic that hit many countries during the early winter of 2020. This wave is still going on to this day and is expected to continue well into 2021 as a third wave, despite recent successes in the development, production and distribution of the long-awaited vaccines.

With various kinds of cyberattacks either on the rise or still going strong, this article seeks to shed some light on arguably the most common form of cybercrime during this pandemic: Covid-19-related “phishing” and “malware” attacks. In doing so, we will both take a look the different kinds of phishing methods perpetrators have come to use and examine the legal implications that come along with them.

While the practical phenomena and legal issues discussed in this paper are primarily based on observations of the current cybercrime scene in Germany, analogous reports from many other countries indicate that law enforcement agencies around the world are confronted with very similar issues (e.g. [7, 21]), showing once more that the challenges and questions posed by the spread of the virus are just as global as the pandemic itself.

## Cyberattacks during the pandemic

In forensic practice, cybercriminals have proven to be very adaptative to the new conditions generated by the Covid-19 pandemic. Out of their commonly known repertoire, two methods have proved to be especially suitable to exploit people’s good faith and their increased need for information and digital interaction during these chronically uncertain times: phishing and malware attacks.

### General modus operandi of phishing and malware attacks

With respect to the phenomenon of phishing, there is a fundamental distinction to be made between the two different crime phases: The phase of data acquisition and the phase of data use.

During the data acquisition phase, the perpetrators try to deceive the potential victim into revealing sensitive data, especially access data for online banking and other payment systems (such as PayPal), either by email or via special websites [1]. In doing so, they regularly pretend to be a supposedly reputable third party, such as a well-known bank or a public authority, by mimicking their corporate identity, style of communication or website design. The perpetrators then request the desired information under a pretext, e.g. to close an existing security gap or to update existing customer data. Such email messages and websites are often designed in a highly professional fashion,^1^ sometimes giving the appearance of authenticity even upon close examination.

Once they follow the perpetrators’ request and transmit the desired data, the second phase begins with the perpetrators trying to use the data for financial gains. This can, for example, be achieved by selling the data, purchasing goods at the expense of the victim or—if the data in question grants access, e.g. to an online banking account—by transferring money from the victim’s account to a third-party account. In order to disguise their own identity, cybercriminals regularly use one of two strategies: They either employ another party to function as a (more or less) unsuspecting money laundering agent, who allows money to be transferred to their private bank accounts and then forward it to the perpetrators for a commission, or they use alias accounts opened under a fake identity (so-called bank drops) [10]. In the case of deliveries of goods, the identity of the actual perpetrators is similarly concealed in order to make prosecution more difficult. In these cases, goods agents are recruited by cybercriminals with a high earning potential. These agents are asked to provide their address for the delivery of the parcels, receive the respective consignment of goods, repackage it, label it and then send it on. This way, the perpetrators secure the loot and exploit it immediately without having to make an overt appearance.

As a variation of the phishing method described above, malware attacks use similar, yet technically often more sophisticated means, especially with respect to the phase of data acquisition. Malware attacks have in large part emerged as a direct reaction to the improvements of cybersecurity concepts within companies. In order to infect the company’s home network with the malware program, the perpetrators usually send fictitious emails^2^ to potential victims from supposedly reputable senders. These emails include an attachment the perpetrator wants the potential victim to open on their device, e.g. an invoice or an order confirmation. As soon as the attached file is opened by its recipient, the malware is installed in the background and begins to record all relevant future activities on the infected device, forwarding all the data needed for the second phase of the crime directly to the perpetrator [6, § 269 para. 15].

### Methodical adaptations during the pandemic

A characteristic feature of the methods described above is that the perpetrators seek to exploit human emotions and traits like trust, curiosity, respect for authority, fear or concerns in order to induce their potential victims to ignore any sense of doubt, precaution and other protective mechanisms (so-called social engineering).^3^ Keeping this in mind, it is not hard to see why a society caught in the middle of a global pandemic is an ideal host for the spread of such criminal practices and why cybercriminals have made increasing use of Covid-19-related issues as a thematic focal point of their attacks.

One example of this is the various methods specifically designed to exploit the financial concerns of many companies facing shutdowns and other restrictions with potentially devastating effects to their businesses. The state police in the German state of Lower Saxony for instance observed a significant number of phishing cases where cybercriminals claimed to be public officials in the service of the Federal Employment Agency, offering unsolicited assistance in applying for public emergency aid granted by the federal government to temporarily reimburse companies for the costs of wages owed to their employees. Under the pretext of requiring specific information in order to proceed with the application, the perpetrators requested different sets of critical data about the company and their employees to be sent to an email address under their control.^4^

Similar to these phishing attacks, cybercriminals have also used malware-based approaches to gain access to data from people and companies in need of public emergency aid. One way in which this was done was sending emails in the name of a public bank known to be administering the distribution of the federal emergency aid funds for small businesses and self-employed entrepreneurs. The message claimed to inform the potential victim of a (supposed) legal duty to report any emergency aid received from the bank to the competent tax authorities using a (malware-infected) document attached to that email.^5^

Another way in which the methods of cybercriminals have been adapted to the pandemic is to take advantage of people’s strong demand for information on the development of the pandemic itself. Some perpetrators for example offered potential victims so-called Coronavirus maps,^6^ disguised malware apps which were supposed to show the current rate of COVID-19 infections in real time. Depending on how the malware app was programmed, it either read out the victim’s sensitive data^7^ or completely locked the infected device and demanded a ransom in order to be unlocked again.^8^

Another popular and quite effective kind of Covid-19-based cyberattack came in the form of so-called man-in-the-middle-attacks,^9^ a term traditionally used to describe schemes where the perpetrator uses specifically designed malware to insert himself between two digital communication partners and to manipulate their data transfer at will. Under the shadow of the current pandemic, however, cybercriminals managed to pull-off this scheme even without the use of sophisticated malware programs, simply by taking advantage of their victims’ existential economic fears. In the German state of North Rhine-Westphalia, cybercriminals created fake websites almost fully mimicking the website of the state’s Ministry of Economics where businesses were supposed to fill out their application forms to gain access to subsidies form the federal emergency fund. Once people had entered the necessary data into the application forms, the perpetrators would simply replace the businesses’ bank details with their own and then forward the complete application to the state authorities responsible for granting the subsidies.^10^ According to initial findings, this method has allowed the perpetrators to gain access to information on 3500–4000 businesses and their applications, potential causing damages in the mid-double-digit millions.

While criminals have thus been quite successful in adapting to the pandemic and using it to their economic advantage, concerns by international law enforcement agencies about other corona-related cybercrimes with potentially more devastating effects seem to have not come true. In Germany at least, authorities have not detected any significant increase in attacks on critical infrastructures,^11^ especially healthcare facilities, during the past several months. While there have been some incidents which do appear to be linked to the pandemic, most notably an attack on the European Medicines Agency,^12^ these attacks do not seem to reflect an overall increase and/or general change of strategy with respect to cyberattacks on critical infrastructures.

## Application of criminal law to pandemic-related cyberattacks

As the experience of the last few months shows, cybercriminals have proven to be as adaptive as they are creative in exploiting the current crisis in their favor. Even though this situation naturally presents law enforcement authorities with some new practical challenges, the legal issues that arise can mostly be resolved adequately by relying on already existing laws and legal methods. The reason for this is that although the thematic focal point of many cyberattacks may have shifted, the perpetrators overwhelmingly fall back on their familiar methodological repertoire when implementing them. For some years now, legislators and courts in most countries have already found appropriate ways to deal with this repertoire under criminal law. The following analysis will exemplify the general legal classification of phishing and malware attacks as the two main forms of pandemic-related cybercrime under German criminal law. However, the basic considerations of this analysis can also be applied to many other legal systems, in particular those of civil law countries whose codified criminal law bears resemblance to the German Criminal Code (StGB).

As explained above,^13^ there are two basic phenomenological phases of phishing: The phase of data acquisition and the phase of data use. This distinction also continues on a legal level and has corresponding consequences for the criminal classification of the perpetrator’s behavior:

The data acquisition phase does not in itself constitute a “fraud” or “computer fraud” (sec. 263 and 263a StGB), since the victim does not suffer any direct financial losses at this point.^14^ However, even the first phase of phishing does usually come along with a number of other criminal offenses. In the case of phishing—irrespective of whether it is carried out by email or by creating a fake website—the first phase constitutes the offense of “preparing to spy out and intercept data” pursuant to sec. 202c StGB, since it is intended to enable access to passwords or other security codes (sec. 202c (1) no. 1 StGB).^15^ If the phishing email gives the impression that the message supposedly comes from a third party that actually exists (as is often the case), this constitutes a “falsification of data with probative value”, which is punishable in Germany under sec. 269 StGB.^16^ If the perpetrators use phishing websites, like the one that mimicked the official website for federal emergency funding in North Rhine-Westphalia, this also constitutes a falsification of data with probative value. If, in addition to this, copyrighted signs are being used in the email or on the fake website, for example the logo of a bank administering Corona subsidies, criminal offenses under trademark and copyright law also come into consideration (see sec. 143 (1), 143a (1) of the German Trademark Law (MarkenG)) and sec. 106 et seq. of the German Copyright Law (UrhG) [15].

If the crime reaches the second phase, the perpetrators usually commit “computer fraud” under sec. 263a StGB [10], e.g. if they use obtained bank account data to make an unauthorized transfer via online banking. At the same time, other criminal offenses will often also be committed, in particular an “unauthorized processing of personal data” (sec. 42 (2) No. 1 of the German Federal Data Protection Law (BDSG)) [3, § 42 15BDSG para. 45, ], as well as (another) falsification of data with probative value (sec. 269 StGB) [10]. If the perpetrator uses the data to apply for Corona emergency aid without authorization, the special offense of “subsidy fraud” under sec. 264 StGB supersedes the general offense of computer fraud under § 263a StGB in Germany.^17^

Similar to phishing, the criminal law assessment of malware attacks is based on two basic factual phases: The first phase of sending and installing the malware program and the second phase of the program being executed. In the first phase, the perpetrators are generally liable to prosecution for “computer sabotage” (sec. 303b (1) no. 2 StGB). Once the program has been installed, further criminal liability depends on its specific mode of operation: If it has been programmed to lock the infected system in order to demand a ransom under the threat of serious harm, e.g. to delete all personal data (so-called ransomware), such an act will fall under the definition of “blackmail” according to sec. 253 (1) StGB,^18^ as well as “illegal data alteration” (sec. 303a (1) var. 2 StGB) [6, § 303a para. 18]. If the malware serves to automatically spy out the victim’s data and send it to the perpetrators, criminal liability for “data espionage” under sec. 202a (1) StGB can be assumed.^19^ In this case scenario, the criminal offenses will often involve a third phase in which the perpetrators use the data to obtain monetary gains. The possible criminal offenses then correspond to those of the second phase of phishing, usually in the shape of computer fraud pursuant to sec. 263a StGB by causing unauthorized (online) bank transfers.

## Conclusion

The last months of the pandemic have shown how incredibly fast cybercriminals were able to adapt to the new circumstances and use them to their advantage. The main reason for this pandemic of cybercrimes appears to be the fact that the perpetrators’ traditional toolbox, in particular phishing and malware methods, proved to be perfectly suited to being reconfigured and applied to a global situation that is as novel as it is unsettling for the population and which has caused widespread emotional and economic distress to many people, often up to the point of threatening their entire livelihoods. With more people spending more time in the digital world, the number of potential victims and opportunities to prey on these victims has also increased. On the bright side, however, cybercrimes and viruses seem to differ in one important way: While potential mutations to Covid-19 always pose a serious threat to our current vaccines and other defense mechanisms become more or less obsolete, adaptations of cybercrimes tend to rely on the same methods prosecutors have come to know and expect. Thus, most countries’ criminal law systems are prepared to deal with the new kinds of cybercrimes and will most likely continue to do so in the future. Covid-19 may be a catalyst for cybercrime, but the legal antidote has luckily already been developed within our existing criminal codes. The real issue with this antidote—much like with its medical counterpart—is its efficient distribution in practice, since cybercriminals are notoriously hard to pin down if they are smart enough to make proper use of the digital world’s various tools to cover their tracks. Thus, prosecutors will have to carefully watch the ways in which cybercriminals adapt to the new environment and constantly stay alert in order to monitor this aspect of the current pandemic as well as possible.

## Footnotes

1 For an account of the development of phishing, see [10]; the specialization of perpetrators has probably also contributed to the increased professionalism, see also [11] on the phenomenon treated under the catchword “crime-as-a-service”.

2 As time goes by, the system is increasingly being infected by exploiting vulnerabilities in victims’ to access fraudulent websites without further individual action by the victim being required (drive-by infection), see also [2], according to whom the infection of a computer can hardly be prevented.

3 MünchKommBGB/*Jungmann* (8th Ed., 2020, § 675l para. 39 [9]); on social engineering in connection with home office in the age of the Corona pandemic, see [5].

4 Announcement by the state office of criminal investigation of Lower Saxony (LKA Lower Saxony) from 02.04.2020 [22].

5 Announcement by the state office of criminal investigation of Lower Saxony of 05.05.2020 [23].

6 The Federal Criminal Police Office in Germany (BKA) warned against such cards as early as April 2020, see BKA communication dated 03.04.2020 [24].

7 BKA communication dated 03.04.2020 [24].

8 Communication of the LKA Lower Saxony of 14.04.2020 [25].

9 In this regard [15, 10, para. 282] speaks more appropriately of “man-in-the-system” in this context.

10 Redaktion beck-aktuell from 14.04.2020, becklink 2016019.

11 BT-Drucks. 19/19676, p. 10 para. 11; this conclusion is also reached by [7], who generally find no corona-related increase in cyberattacks in the United States.

12 See The Guardian from 09.12.2020 [26].

13 See under Sect. 2.1.

14 [1, 15] with further details; other assessment by [4], who considers the data itself to be of financial value, thus assuming that “risk-based damage” (Gefährdungsschaden) does immediately occur.

15 [10]; MünchKomm StGB/*Erb* (3rd Ed., 2019, § 269 para. 34 [8]);[15]; in general also [16].

16 MünchKomm StGB/*Erb* (3rd Ed., 2019, § 269 para. 33 [8]); [6, § 269 para. 15].

17 On the criminal liability of subsidy fraud in connection with the Corona emergency aid, see [13].

18 [14, § 253 para. 11], on “digital extortion” see also [12].

19 For a detailed discussion, see [10].
